# Albumin Alters the Conformational Ensemble of Amyloid-β by Promiscuous Interactions: Implications for Amyloid Inhibition

**DOI:** 10.3389/fmolb.2020.629520

**Published:** 2021-02-23

**Authors:** Huisi Xie, Cong Guo

**Affiliations:** Department of Physics and International Centre for Quantum and Molecular Structures, College of Sciences, Shanghai University, Shanghai, China

**Keywords:** alzheimer’s disease, amyloid-beta, serum albumin, conformational ensemble, solute tempering, promiscuous interactions

## Abstract

Human serum albumin (HSA) is a key endogenous inhibitor of amyloid-β (Αβ) aggregation. In vitro HSA inhibits Aβ fibrillization and targets multiple species along the aggregation pathway including monomers, oligomers, and protofibrils. Amyloid inhibition by HSA has both pathological implications and therapeutic potential, but the underlying molecular mechanism remains elusive. As a first step towards addressing this complex question, we studied the interactions of an Aβ42 monomer with HSA by molecular dynamics simulations. To adequately sample the conformational space, we adapted the replica exchange with solute tempering (REST2) method to selectively heat the Aβ42 peptide in the absence and presence of HSA. Aβ42 binds to multiple sites on HSA with a preference to domain III and adopts various conformations that all differ from the free state. The β-sheet abundances of H14-E22 and A30-M33 regions are significantly reduced by HSA, so are the β-sheet lengths. HSA shifts the conformational ensemble towards more disordered states and alters the β-sheet association patterns. In particular, the frequent association of Q15-V24 and N27-V36 regions into β-hairpin which is critical for aggregation is impeded. HSA primarily interacts with the latter β-region and the N-terminal charged residues. They form promiscuous interactions characterized by salt bridges at the edge of the peptide-protein interface and hydrophobic cores at the center. Consequently, intrapeptide interactions crucial for β-sheet formation are disrupted. Our work builds the bridge between the modification of Aβ conformational ensemble and amyloid inhibition by HSA. It also illustrates the potential of the REST2 method in studying interactions between intrinsically disordered peptides and globular proteins.

## Introduction

The pathogenesis of Alzheimer’s disease (AD) is tightly correlated with the abnormal aggregation of amyloid-β (Aβ) in the central nervous system (CNS). Numerous endogenous proteins interacting with Aβ can modulate its amyloidogenic process (Bohrmann et al., 1999; Han et al., 2016). Human serum albumin (HSA), the most abundant protein in blood, has been recognized as an inhibitor of Aβ aggregation (Biere et al., 1996; Bohrmann et al., 1999; Kuo et al., 2000; Ezra et al., 2016). It binds Aβ and facilitates Aβ efflux from the cerebrospinal fluid (CSF) to plasma (Boada et al., 2020). Reduced serum albumin levels are associated with increasing cognitive impairment in AD patients (Yamamoto et al., 2014). Moreover, a phase IIb/III trial using plasma exchange with albumin replacement has presented initial encouraging results (Boada et al., 2020). In vitro, substantial evidence suggests that HSA inhibits Aβ aggregation and binds multiple species along the aggregation pathway which include monomers, oligomers, and protofibrils (Milojevic et al., 2007; Milojevic et al., 2009; Milojevic and Melacini, 2011; Stanyon and Viles, 2012; Algamal et al., 2013; Milojevic et al., 2014; Wang et al., 2016; Choi et al., 2017; Algamal et al., 2017; Bode et al., 2018). Despite the biological and therapeutic significance of HSA-Aβ interactions, the underlying mechanism is not fully understood. Molecular dynamics (MD) simulations hold great potential to contribute to solving the puzzle. However, with conventional MD, it is challenging to adequately sample the conformational space of the Aβ-HSA complex due to the intrinsic disorder of Aβ and the large system size. The present work reports the adaption of an enhanced sampling method called replica exchange with solute tempering (REST2) (Wang et al., 2011) to study the interactions of monomeric Aβ with HSA.

Aβ is a 36-43-residue peptide derived from the amyloid precursor protein (Nasica-Labouze et al., 2015). The two common isoforms are the 40-residue Aβ40 and 42-residue Aβ42, with the latter having two extra residues (I41-A42). Although Aβ40 is more abundantly produced, Aβ42 is more disease relevant as it is more abundant in amyloid plaques and shows a greater tendency to aggregate in vitro (Nasica-Labouze et al., 2015). The amino acid sequence of Aβ42 can be divided into four regions according to hydrophobicity: the hydrophilic N-terminal D1-K16 region that is comprised of 6 charged residues and 3 histidines, the central hydrophobic core (CHC) region L17-A21, the hydrophilic central region E22-G29, and the hydrophobic C-terminal region A30-A42. Monomeric Aβ is classified as an intrinsically disordered peptide (IDP), but solution nuclear magnetic resonance (NMR) experiments have detected transient β-sheet structures, especially in the CHC, I31-V36, and V39-I41 regions (Hou et al., 2004). β-hairpin conformation with two legs at residues L17-D23 and A30-V36 was stabilized by the amyloid inhibitor protein Z_Αβ3_, indicating an important role of the β-hairpin structure in fibrillization (Hoyer et al., 2008). Different from monomers, Aβ fibrils are featured by in-register parallel cross-β sheet structures. Recently, several groups have solved atomic resolution structures of Aβ42 fibrils with advanced solid-state NMR and cryo-electron microscopy (cryo-EM) techniques (Xiao et al., 2015; Colvin et al., 2016; Wälti et al., 2016; Gremer et al., 2017). In these structures, the N-terminal region is disordered or partially ordered while the other regions are arranged into 3 or 4 β-strands linked by loops, which results in an overall S-shape. Especially, residues in the CHC region and the C-terminal region constitute the cross-β structures in all structures, reinforcing their critical roles in aggregation as have been established by many studies (Liu et al., 2004; Williams et al., 2004; Bernstein et al., 2005).

The aggregation process of Aβ is described by a nucleation-condensation polymerization model, which involves a lag phase for nucleation, a subsequent elongation phase for the rapid growth of oligomers and protofibrils into fibrils, and a final plateau phase. Though Aβ peptides circulate in CSF and in blood at similar concentrations of 0.1–0.5 nM (Stanyon and Viles, 2012), amyloid plaques were only found in CNS. It is primarily attributed to the fact that ∼90% plasma Aβ is sequestered by HSA which has a concentration of 640 μM in plasma as opposed to a remarkably low level of 3 μM in CSF (Biere et al., 1996). In vitro, HSA at physiological concentrations significantly increased the lag phase time and decreased the total amount of amyloid fibers (Stanyon and Viles, 2012). A 35-residue segment in domain III retained the inhibitory effect of HSA (Picón-Pagès et al., 2019) while natural HSA ligands negated such effect (Bode et al., 2018). HSA interfered with different stages of aggregation and targeted multiple species including monomers, oligomers, and protofibrils with increasing affinities (Wang et al., 2016; Algamal et al., 2017). Although the molecular mechanism underlying the protective inhibition of Aβ aggregation by HSA has not been fully elucidated, these studies consistently indicate a role of monomeric Aβ-HSA interactions in the process, which also lay the foundation for high-order interactions between Aβ oligomers/protofibrils and HSA. Therefore, revealing the interaction mechanism of monomeric Aβ with HSA is essential for understanding the amyloid regulation by HSA.

Many experiments have been devoted to studying the monomeric Aβ-HSA interactions but current understanding of this issue is still limited due to certain inconsistency in the literature. HSA was found to bind monomeric Aβ at a stoichiometric ratio of 1:1 (Kuo et al., 2000). It is agreed that the monomeric Aβ-HSA interactions are weak. However, very different disassociation constants (*K*_*d*_) ranging from submicromolar to submillimolar have been reported (Rózga et al., 2007; Costa et al., 2012; Wang et al., 2016; Algamal et al., 2017). Aβ40 and Aβ42 have different affinities to HSA whereas the order of the two is a subject of debate (Algamal et al., 2017; Litus et al., 2019). Molecular-level characterization of Aβ binding to HSA has also been provided. Saturation transfer difference NMR experiments by Algamal et al. have identified the C-terminal region of Aβ as the primary interaction site with HSA (Algamal et al., 2017). With mass spectrometry and small-angle X-ray scattering, Choi and coworkers found that HSA predominantly captured a single Aβ monomer at the groove between domains I and III, resulting in a structural change of Aβ from a random coil to an α-helix but no structural variations of HSA (Choi et al., 2017). Contradictorily, a more recent study reported that domain II contained the primary binding sites for Aβ monomers (Ishima et al., 2020). The above discrepancies could be due to different Aβ sample preparation procedures and buffer conditions which are shown to influence the Aβ-HSA interactions (Litus et al., 2019) and the presence of Aβ oligomers in the sample resulting from the intrinsic propensity of Aβ to aggregate. These factors bring challenges to experimental measurements on the monomeric Aβ-HSA interactions. Several questions remain open: 1) a comprehensive characterization of the Aβ conformations and binding sites in the complex with HSA is still lacking, which is essential for understanding the interaction mechanism; 2) it is unknown how such information is related to amyloid inhibition.

MD is a powerful tool to probe the molecular mechanisms at the atomic level through investigating conformational ensembles of biomolecules. Previously using conventional MD simulations, we found that domain III was the primary target for Aβ binding and that fatty acids interfered with Aβ binding to HSA by quenching the conformational flexibility of the latter (Guo and Zhou, 2019). However, we failed to capture any possible Aβ conformational transitions upon binding to HSA, probably due to the relatively short simulation time and Aβ as an IDP possessing a flat free energy surface. On this issue, enhanced sampling methods are needed, among which replica exchange molecular dynamics (REMD) (Sugita and Okamoto, 1999) has been widely used to study Aβ peptides (Rosenman et al., 2016; Man et al., 2017) and other IDPs (Guo et al., 2015). In REMD, multiple replicas of a system are simulated at different temperatures simultaneously and neighboring replicas are attempted to exchange periodically using the Metropolis criterion. A random walk of replicas in the temperature space allows them to escape local minimum. However, the use of REMD to large systems such as the Aβ-HSA complex (>620 residues) is computationally restricted by the large number of replicas required to cover a wide temperature range with reasonable exchange probabilities.

As an alternative, the replica exchange with solute tempering (REST) method has been developed (Liu et al., 2005) and later modified in REST2 (Wang et al., 2011) to improve sampling efficiency. It has been successfully applied to the conformational sampling of IDPs (Côté et al., 2015; Rossetti et al., 2016; Smith et al., 2016; Han et al., 2017; Lee and Chen, 2017; Hicks and Zhou, 2018). REST2 is a new form of Hamiltonian replica exchange method wherein all replicas are simulated at the same temperature *T*_0_ albeit on different deformed potential energy surfaces. With delicate energy scaling, exchange probability between two replicas is exclusively determined by protein-related energy terms that involve a small number of atoms, not by the energy of a large number of solvent molecules. Consequently, the number of replicas can be reduced four to five times without changes in the temperature range (Smith et al., 2016). Another important consequence is that part of the solute instead of all solute atoms can be chosen for scaling to achieve enhanced sampling. For example, it has been used to sample the conformations of a disordered loop in a globular protein (Pang and Zhou, 2015). This feature is perfectly suited for exploring the conformational ensemble of Aβ in the large complex with HSA, whereby Aβ is highly dynamic while HSA experiences little conformational changes (Choi et al., 2017).

Herein, we have employed the REST2 protocols to study the interactions of the more toxic Aβ42 monomer with HSA. By choosing Aβ42 atoms for scaling, we can use the same number of replicas to achieve enhanced sampling of Aβ42 with and without HSA. Simulations of the isolated Aβ42 peptide yield consistent secondary structure contents with previous REMD studies (Rosenman et al., 2016), demonstrating the applicability of the REST2 protocols. Aβ42 binds to five major sites on the HSA surface with a preference to domain III, consistent with our previous work (Guo and Zhou, 2019). The binding site at the cleft of domains I and III is similar to the one reported by ion mobility mass spectrometry (Choi et al., 2017). Aβ42 adopts different conformations at different binding sites, which in general are less β-sheet-rich and contain shorter β-strands than the free state. HSA significantly suppresses the β-sheet propensities of the H14-E22 and A30-M33 regions and alters the intrapeptide interaction patterns as well. Particularly the interactions between the Q15-V24 region and the N27-V36 region which are dominant in the free state are disrupted by HSA. Aβ42 interacts with HSA primarily via the N-terminal charged residues and the K28-M35 segment. An interaction mechanism is proposed wherein Aβ42 promotes promiscuous interactions with HSA that conflict with intrapeptide interactions curial for β-sheet formation. Implications of our findings in amyloid inhibition are also discussed.

## Materials and Methods

### System Preparation

**FIGURE 1:**
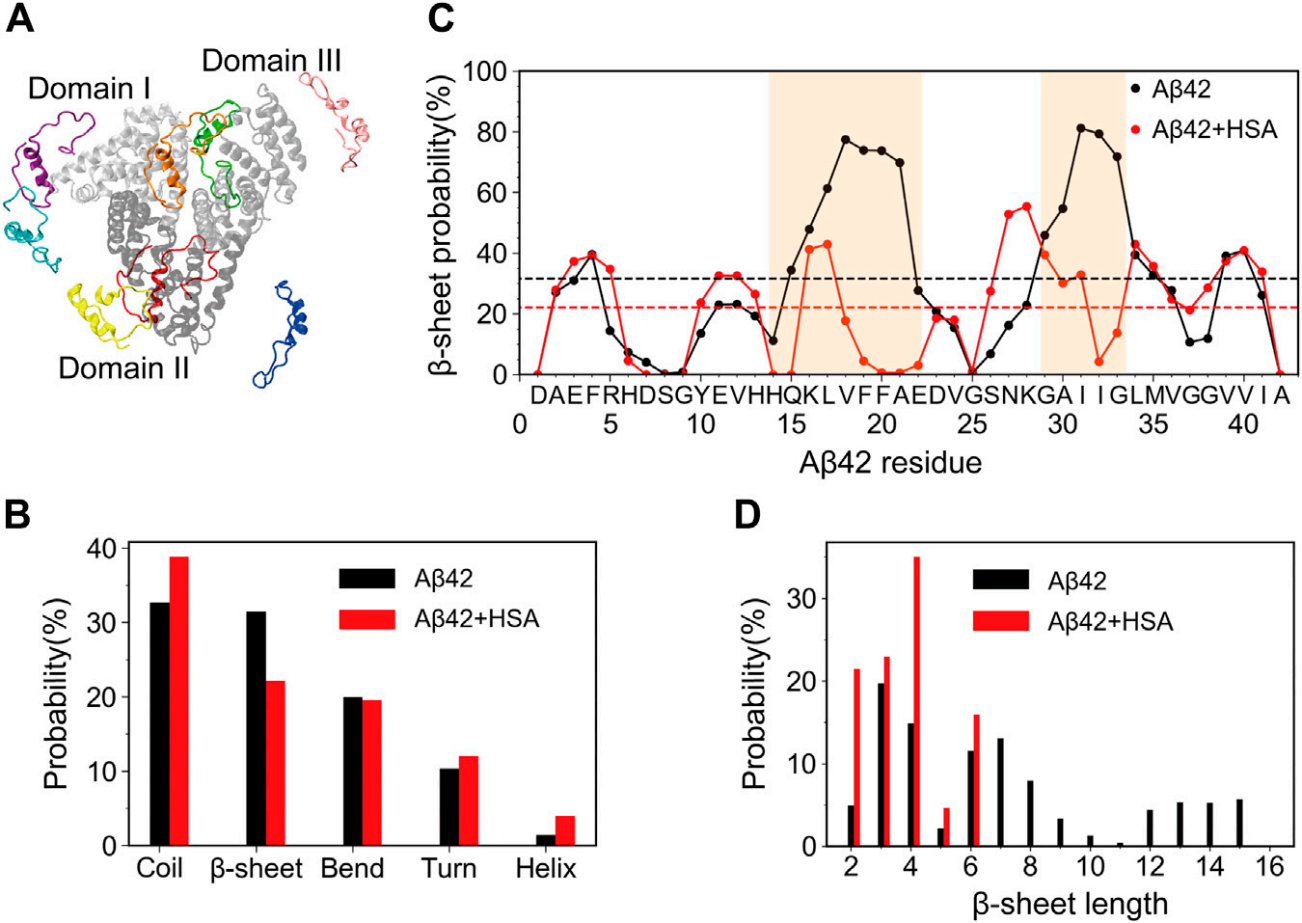
Starting structures of simulations for Aβ42 with HSA and secondary structure changes of Aβ42 upon binding to HSA. **(A)** Superimposition of 8 Aβ42 (in color) starting positions around HSA. Domains I, II, and III of HSA are shown in silver, gray, and light gray, respectively. **(B)** The average probability of each secondary structure content. **(C)** Residue-specific β-sheet probability. The average value of each curve is shown as a horizontal dashed line. Regions (H14-E22 and G29-G34) that display significant changes are highlighted by brown shading. **(D)** Histograms of β-sheet lengths of Aβ42 in the two systems.

The sequence of Aβ42 is DAEFRHDSGY^10^ EVHHQKLVFF^20^ AEDVGSNKGA^30^ IIGLMVGGVV^40^ IA. The starting structure of Aβ42 was built upon the NMR structure of Aβ40 in aqueous solution (PDB 2LFM) (Vivekanandan et al., 2011) by adding the two C-terminal residues (I41-A42) with PyMol (DeLano, 2002). HSA is a 585-residue protein and consists of three homologous domains I to III (Figure 1A). Each domain can be further divided into subdomains a and b. The initial coordinates of HSA were taken from its crystal structure (PDB 1AO6) (Sugio et al., 1999). Two systems were simulated, the Aβ42 monomer alone (Aβ42) and in the presence of HSA (Aβ42 + HSA). The Aβ42 + HSA system contained one Aβ42 molecule and one HSA molecule, for which 8 different initial configurations (Figure 1A) were generated by randomly placing the Aβ42 peptide at different positions 10 Å away from HSA. Each initial configuration seeded two replica simulations.

### Simulation Setup

We performed all simulations using the GROMACS 2018.1 software package (Abraham et al., 2015) patched with the PLUMED plug-in (version 2.4.2) for REST simulations (Bussi, 2014; Tribello et al., 2014). GPU acceleration (Páll and Hess, 2013) was used to increase computation performance. The Amber99sb-ILDN (Lindorff-Larsen et al., 2010) force field and the TIP3P water model were used. For both Aβ42 and Aβ42 + HSA, the solute was energy minimized in vacuum first and then solvated in a dodecahedron box with a minimal distance of 10 Å from the box boundaries. Counterions were added to neutralize the net charge of proteins and generate a salt concentration of 150 mM. The whole system was heated gradually to 300 K in 200 ps. Then, it was equilibrated for 200 ps under an NVT ensemble and for another 200 ps under an NPT ensemble. During the whole equilibration process, protein heavy atoms were restrained. In the final production runs, these restraints were removed and all protein bonds were restrained by LINCS (Hess et al., 1997). The Particle Mesh Ewald method (Darden et al., 1993) with a real-space cut-off of 10 Å was used to calculate long-range electrostatic interactions. Temperature was maintained at 300 K by the velocity rescaling method (Bussi et al., 2007). Pressure was maintained at 1 bar by the Parrinello-Rahman coupling method (Parrinello and Rahman, 1981; Nosé and Klein, 1983). The simulation time step was 2 fs. Snapshots were saved every 10 ps. More details about REST2 simulation parameters are given below.

### Details of REST2 Protocol

The REST2 method was used to enhance the sampling of the Aβ42 peptide. In REST2, the total potential energy of a system is decomposed into three components: the protein intramolecular energy *E*_*pp*_, the interaction energy between protein and solvent *E*_*pw*_, and the self-interaction energy between solvent molecules *E*_*ww*_. For each replica, its potential energy is

$$E=\lambda E_{pp}+\sqrt{\lambda }E_{pw}+E_{ww}.$$

Scaling is limited to the first two terms and all replicas are assigned different scaling factors *λ* ranging from 0 to 1. Enhanced sampling is achieved by equivalently heating protein to a higher effective temperature *T*_0_/*λ* while the solvent molecules remain cold at *T*_0_. For both Aβ42 and Aβ42 + HSA, all atoms of the Aβ42 peptide were selected as the “hot” solute region; the other atoms were kept unperturbed which were equivalently treated as the “solvent” region. In different replicas, Aβ42-Aβ42 and Aβ42-other interactions were scaled to generate an effective temperature ladder for the “hot” region, while the “solvent” temperature remained a constant. We used 16 replicas at the effective temperatures exponentially spaced between 300 and 600 K. The effective temperature ladder was 300.0, 314.1, 328.9, 344.8, 361.0, 377.8, 395.8, 414.4, 434.2, 454.5, 476.2, 498.3, 522.6, 547.4, 572.5, and 600.0. Exchange between neighboring replicas was attempted every 2 ps. The average exchange rates for the two systems are the same, 33.2% for Aβ42 and 32.8% for Aβ42 + HSA. Each replica simulation lasted 800 ns for Aβ42 and 500 ns for Aβ42 + HSA. For both systems, the last 200 ns from the unscaled replica (i.e., at 300 K) was used for analysis.

### Analysis

All analyses were carried out with built-in tools in GROMACS and our in-house-developed codes. Secondary structures of Aβ42 were determined by the DSSP (Kabsch and Sander, 1983) program. The cluster analysis of Aβ42 conformations was performed with gmx cluster in GROMACS using a backbone root-mean-square deviation (RMSD) cut-off of 0.2 nm. The binding propensity of one residue in one protein was defined as the percentage of snapshots in which it was in contact with the partner protein. A contact was defined when two heavy atoms lie within 5.4 Å. For each snapshot, the Aβ42 binding pose was characterized by the position of Aβ42 relative to HSA, which was calculated as the center-of-mass coordinates of Aβ42 after superimposing HSA to the starting structure using backbone atoms. All poses sampled in the last 200 ns were partitioned into clusters by the DBSCAN algorithm (Ester et al., 1996). A salt bridge is considered to be formed if the distance between any of the oxygen atoms of acidic residues and the nitrogen atoms of basic residues is within 4 Å. All structure figures were prepared in VMD (Humphrey et al., 1996).

## Results

### Convergence of Simulations

We carried out comparative REST2 simulations of Aβ42 with and without HSA so as to provide atomic-level insight on Aβ42-HSA interactions, with a focus on the effect of HSA on Aβ42 conformational ensemble and binding properties of Aβ42. Two systems are denoted by Aβ42 + HSA and Aβ42, respectively. By taking the advantage that the REST2 method can heat a part of the system, we selectively enhanced the sampling of Aβ42 conformational ensemble with affordable computation cost. A 700/500 ns REST2 simulation was performed for Aβ42/Aβ42 + HSA, which led to an accumulative simulation time of 11.2/9 μs. Throughout each of the two simulations, each of the 16 replicas visited all of the 16 effective temperatures. The percentages of dwell time of 16 replicas at each effective temperature fluctuate around 6.25% with standard deviations at 1∼4% for Aβ42 and at 2∼6% for Aβ42 + HSA (Supplementary Figures S1A,B). It indicates sufficient exchanges between replicas and thus verifies the sampling efficiency. Furthermore, the convergency of simulations was checked by comparing the radius of gyration (Rg) and the secondary structure probabilities of Aβ42 in different time intervals from the unscaled replica (i.e., 300 K). For both systems, the distribution curves of Rg in two independent 100 ns time intervals of the last 200 ns overlap well with each other (Supplementary Figures S1C,D); the probabilities of each secondary structure content in two different time intervals are the same (Supplementary Figures S1E,F). Moreover, secondary structures of Aβ42 are consistent with previous REMD simulations which started from extended coils (Rosenman et al, 2016), evidencing the insensitivity of simulation results to the initial conformation. These results demonstrate that two REST2 simulations have reasonably converged in the last 200 ns.

Initially, the Aβ42 peptide was randomly placed at 8 different positions 10 Å away from HSA. At the effective temperature of 300 K, Aβ42 diffuses onto the surface of HSA within 50 ns and basically remains in a bound state until 500 ns. Disassociation of Aβ42 from HSA is observed but the frequency is extremely low. Especially in the last 200 ns, Aβ42 is disassociated from HSA in only 4% of total frames. With the increase of effective temperature, the binding probability of Aβ42 to HSA decreases. Above 414 K, Aβ42 is bound to HSA in 34∼76% of total frames. During simulations, HSA displayed an average backbone RMSD at 3.5 Å at both low and high temperatures, justifying our assumption that HSA has little conformational changes upon Aβ42 binding. Root-mean-square fluctuations (RMSFs) of HSA residues do not change with temperatures (Supplementary Figure S2). Large conformational changes of HSA are not accessible by our simulations. Structural stabilities of HSA probably account for the high binding probabilities of Aβ42 at high temperatures. Only data from the unscaled replica (i.e., 300 K) are meaningful for analysis, because in the other replicas, the system evolves on a deformed energy surface. Unless specified, all results below are based on data of the last 200 ns at 300 K, during which it is fair to consider that Aβ42 remains bound to HSA.

### HSA Reduces the β-Sheet Abundance of the H14-E22 and A30-G33 Regions of Aβ42 and Prevents Formation of Long β-Strands

We first analyzed the influence of HSA on the secondary structures of Aβ42. The average probability of each secondary structure (including coil, β-sheet, bend, turn, and helix) was calculated. As shown in Figure 1B, the isolated Aβ42 peptide mainly adopts random coil (32.7%) and β-sheet (31.5%) structures, in accordance with its intrinsically disordered nature. Bend and turn contents are a little lower (20.1% and 10.3%) while the helix content (1.4%) can be neglected. These results are similar to those obtained by circular dichroism (CD) spectroscopy (27% β-sheet and 6% helix) (Fezoui and Teplow, 2002) and previous REMD simulations using the same force field (∼36% coil, ∼26% β-sheet, ∼19% bend, ∼16% turn, and <3% helix) (Rosenman et al., 2016). Upon binding to HSA, the β-sheet content is significantly reduced to 20.7%, whereas the coil content increases to 40.5% and the helix content slightly increases to 4.0%. The increase of helix propensities upon complexation with HSA was also detected by previous CD experiments (Choi et al., 2017). The bend and turn contents do not change much, which are 19.9% and 12.0%, respectively.

To elaborate the apparent changes of the β-sheet abundance, we show the residue-specific β-sheet probabilities of Aβ42 with and without HSA in Figure 1C. For each system, the average β-sheet probability is indicated by a horizontal dashed line. For the isolated Aβ42 peptide, three continuous segments form β-sheets, which include two long stretches spanning residues Y10-V24 and S26-I41 and a short stretch covering the N-terminal residues A2-H6. Residues Q15-A21 and G29-M35 exhibit relatively high β-sheet propensities. The former covers the CHC region and the latter belongs to the C-terminal region. We recall that both regions are critical for fibrillization (Liu et al., 2004; Bernstein et al., 2005). Besides, residues E3-F4 and V39-V40 display above-average β-sheet probabilities. Similar β-sheet profiles were reported by previous REMD simulations of the Aβ42 monomer (Rosenman et al., 2016) and dimer (Man et al., 2017). Our results are also consistent with NMR experiments which detected β-strands in the CHC region, residues I31-V36 and V39-I41 (Hou et al., 2004).

In the presence of HSA, the above-mentioned β-regions are preserved to some extent, but pronounced changes occur to two continuous β-segments spanning Y10-V24 and S26-I41. The first region splits into three short ones, Y10-H13, H16-F19, and E22-V24. Particularly, residues H14-E22 suffer the greatest reduction of β-sheets with all β-sheet probabilities falling below the average. The S26-I41 region splits as well at I32. The β-sheet probabilities of residues A30-G33 are also significantly decreased. The discontinuous β-regions in the presence of HSA imply that the length of β-sheets should vary from that without HSA. Therefore, we plotted the histograms of β-sheet lengths of Aβ42 in the two systems (Figure 1D). Without HSA, the β-sheet length of Aβ42 ranges from 2 to 15. Both short β-stands (3–4 residues) and long β-strands (6–8 or 12–15 residues) have relatively high probabilities. With HSA, Aβ42 is more prone to form short β-strands composed of 2–4 and 6 residues; longer β-strands disappear.

Overall, HSA suppresses the β-sheet formation of Aβ42, in line with its inhibitory effect on Aβ fibrillization (Stanyon and Viles, 2012). Not only are the β-sheet propensities at residues H14-E22 and A30-G33 significantly reduced, but also the β-sheet length is much shorter in the presence of HSA. Changes in secondary structures hint at different tertiary structures of Aβ42 in two systems.

### HSA Shifts the Conformational Ensemble of Aβ42 Towards Less β-Sheet-Rich States and Modifies the β-Sheet Associations

**FIGURE 2:**
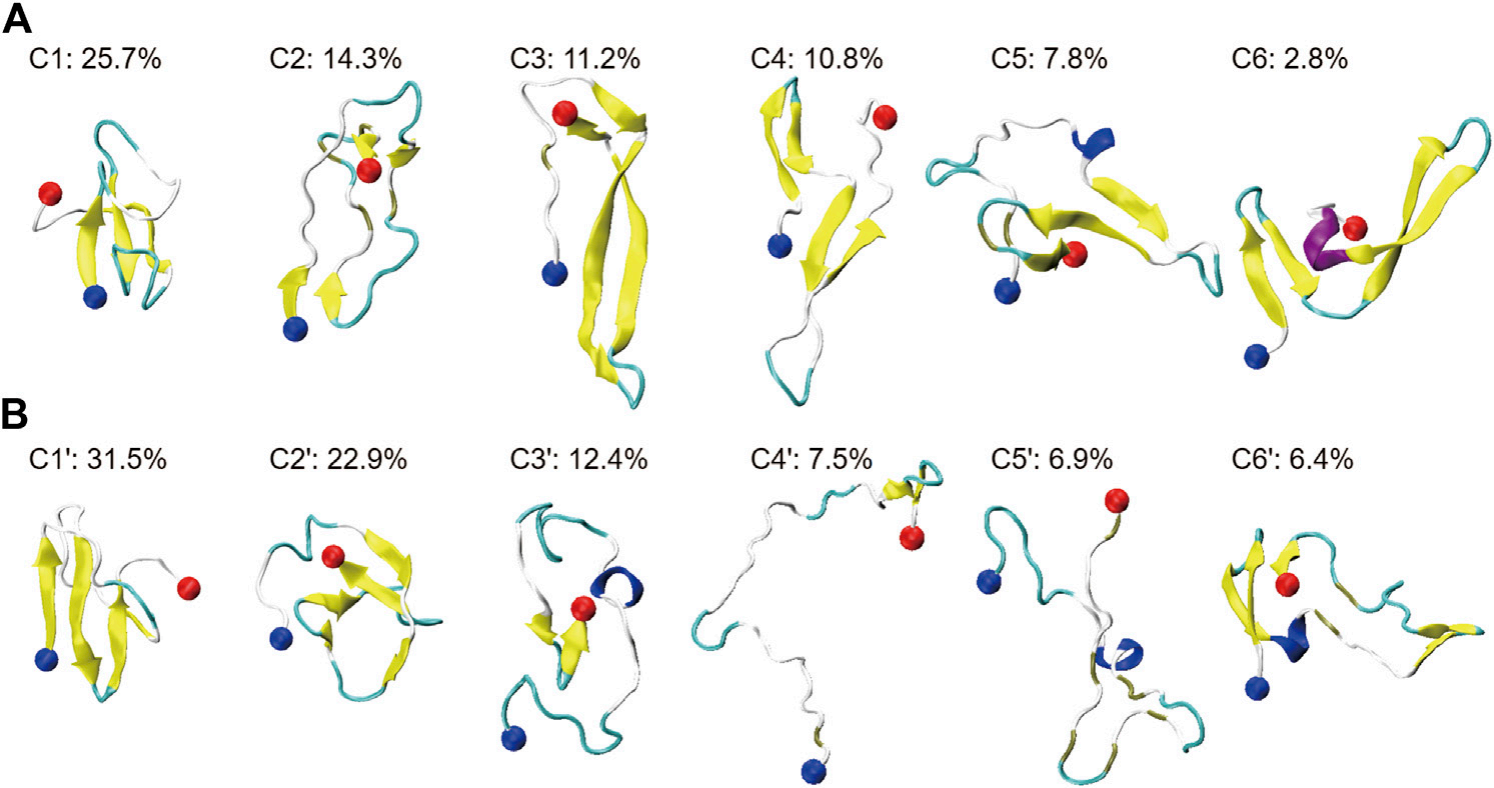
Cluster analysis on the conformational ensemble of the Aβ42 peptide in two systems: **(A)** Aβ42 and **(B)** Aβ42 + HSA. For two systems, representative conformations of the Aβ42 molecule in the top six most-populated clusters are shown as well as the corresponding population of each cluster. The blue and red balls refer to the Cα atoms of the N- and C-terminal residues (D1 and A42), respectively.

To investigate the influence of HSA on the conformational ensemble of Aβ42, we clustered Aβ42 conformations using a backbone RMSD cut-off of 0.2 nm. For Aβ42 and Aβ42 + HSA, 679 and 181 clusters are found, respectively. Representative conformations of the top six most-populated clusters and the corresponding populations are shown in Figure 2 (C1–C6 for Aβ42, C1′–C6′ for Aβ42 + HSA). These clusters account for 73 and 88% of the total snapshots of Aβ42 and Aβ42 + HSA, respectively. For both systems, conformations in the remaining clusters resemble those in the top 6 clusters, as judged by the residue-specific β-sheet probabilities (Supplementary Figure S3). The β-sheet structures in the G29–G37 region are further suppressed by HSA in the remaining clusters of Aβ42 + HSA. Thus, the remaining clusters which all have populations below 1% are omitted here. The smaller number of clusters and the larger proportion of the top 6 clusters reflect that the structural diversity of Aβ42 in the presence of HSA is less pronounced than the isolated form.

Without HSA, the conformational ensemble of Aβ42 is featured by β-sheet-rich structures. The most populated conformation contains a three-stranded β-sheet structure, which also appears in C5. Meanwhile, β-hairpin structures are frequently observed in C3, C4, and C6. Disordered structures are only observed in C2, which contain two short β-hairpins at the N- and C-terminus. In the presence of HSA, even though C1′, C2′, and C6′ are still β-sheet-rich, the conformational ensemble is shifted towards more disordered states. Conformations in C3′, C4′, and C5′ are dominated by extended coils. Among them, the conformation of C4′ is the most extended. In addition, short helices are observed in C3′, C5′, and C6′, accounting for 25.7% of total snapshots. For the isolated Aβ42, helical structures are observed in C5 and C6 with a total percentage of 10.6%. It is consistent with the slight increase of helix content.

**FIGURE 3:**
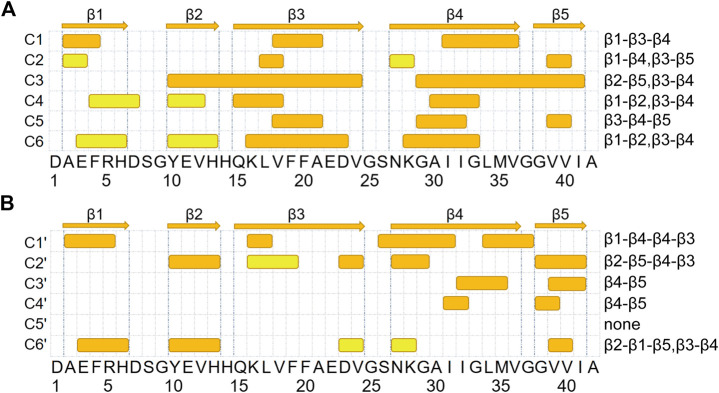
β-sheet associations for Aβ42 and Aβ42 + HSA are displayed in **(A, B)**, respectively. β-strands formed in the representative conformations of the top six most-populated clusters are shown with colored strips. The corresponding snapshot of each conformation is shown in Figure 2. β-strands that associate into the same β-sheet are paired with the same color. At the top, five β-segments are represented by arrows.

To better characterize the tertiary structures, we illustrate the β-strands and β-sheet associations in each representative conformation in Figure 3. β-strands are represented with strips and those assembling into one β-sheet are paired with the same color. We partitioned the whole sequence into 5 conserved β-regions by grouping residues that form β-strands in more than 2 clusters of Aβ42 (C1 to C6) or Aβ42 + HSA (C1′ to C6′). They are identified as follows: A2-H6 (β1), Y10-H13 (β2), Q15-V24 (β3), N27-V36 (β4), and G38-I41 (β5). As have been reported by previous simulations (Song et al., 2015; Man et al., 2017), the extra two C-terminal residues of Aβ42 stabilize an additional β-strand spanning G38 to I41 that is absent in Aβ40. One conformation differs from another in β-regions and the way they assemble into β-sheets. Therefore, we listed the composition of β-regions for each conformation and used a dash character to represent the hydrogen bonding connection. Interestingly, the five β-regions, consistent with previous MD simulations of the Aβ42 monomer (Song et al., 2015; Rosenman et al., 2016) and dimer (Man et al., 2017), overlap well with those in Aβ42 fibrils (Supplementary Figure S5) (Xiao et al., 2015; Colvin et al., 2016; Wälti et al., 2016; Gremer et al., 2017). The best match is with the fibril structures determined by cryo-EM (Gremer et al., 2017), wherein four β-segments are at A2-G9, E11-A21, N27-L34, and V39-I41, respectively, and the second segment combines β2 and β3 here. It implies that β-sheet motifs of the Aβ42 monomer are closely related to the fibrillization process.

For the isolated Aβ42 peptide, the most frequently occurring β-regions are Q15-V24 (β3) and N27-V36 (β4), consistent with the fact that the two regions have the highest β-sheet propensities (Figure 1C). The other three β-regions are also observed in multiple clusters, but the corresponding β-sheet lengths are much shorter (2–4 residues). Furthermore, the most frequent association is between Q15-V24 (β3) and N27-V36 (β4) as well, which appears in all clusters except C2. In C1 and C5, β3 and β4 form a three-stranded β-sheet together with an additional β-strand. REMD simulations of the Aβ42 dimer also reported the similar β-hairpin (CHC and A30-V36) and the three-stranded β-sheet in C5 (L17-A21, A30-V36, and V39-I41) (Man et al., 2017). In C3, C4, and C6, β3 and β4 form a β-hairpin. Τhe Α2−Η6 (β1) region primarily associates with Q15-V24 (β3) as in C1 and also has certain probabilities to associate with Y10-H13 (β2) as in C4 and C6. Τhe V39-I41 (β5) region at the C-terminus mainly associates with Y10-H13 (β2) as in C3 and with N27-V36 (β4) as in C4 and C6. Note that C4 and C6 share the same β-sheet association pattern (β1-β2, β3-β4) but the relative orientations of the resulting two β-hairpins are different. From these data, we conclude that the association of Q15-V24 with N27-V36 (i.e., β3-β4) serves as a core of β-sheet-rich conformations. Consistently, the β-hairpin formed by residues K16-E22 and G29-M35 has been suggested as a basic monomeric unit for the aggregation process (Abelein et al., 2014).

Upon binding to HSA, the β-sheet association of each cluster is different from any of clusters C1 to C6. Though the β3-β4 association is frequently observed in C1′, C2′, and C6′, the β3 strands are much shorter. Moreover, the association between β4 and β5 is also frequent, which occurs in C2′, C3′, and C4′. Meanwhile, two new associations emerge. The first is between two β-segments within the β4 region (i.e., β4-β4) in C1′. The second is between β1 and β5 in C6′. In contrast, the associations of β3 with β1 and β5 observed in C1 and C2, respectively, disappear. To sum up, HSA impairs associations of the Q15-V24 region with the rest, promotes associations of the N27-V36 region with the C-terminus, and induces new associations within the N27-V36 region and between N- and C-terminal β-regions.

### HSA Modifies Intrapeptide Interaction Patterns of Aβ42

**FIGURE 4:**
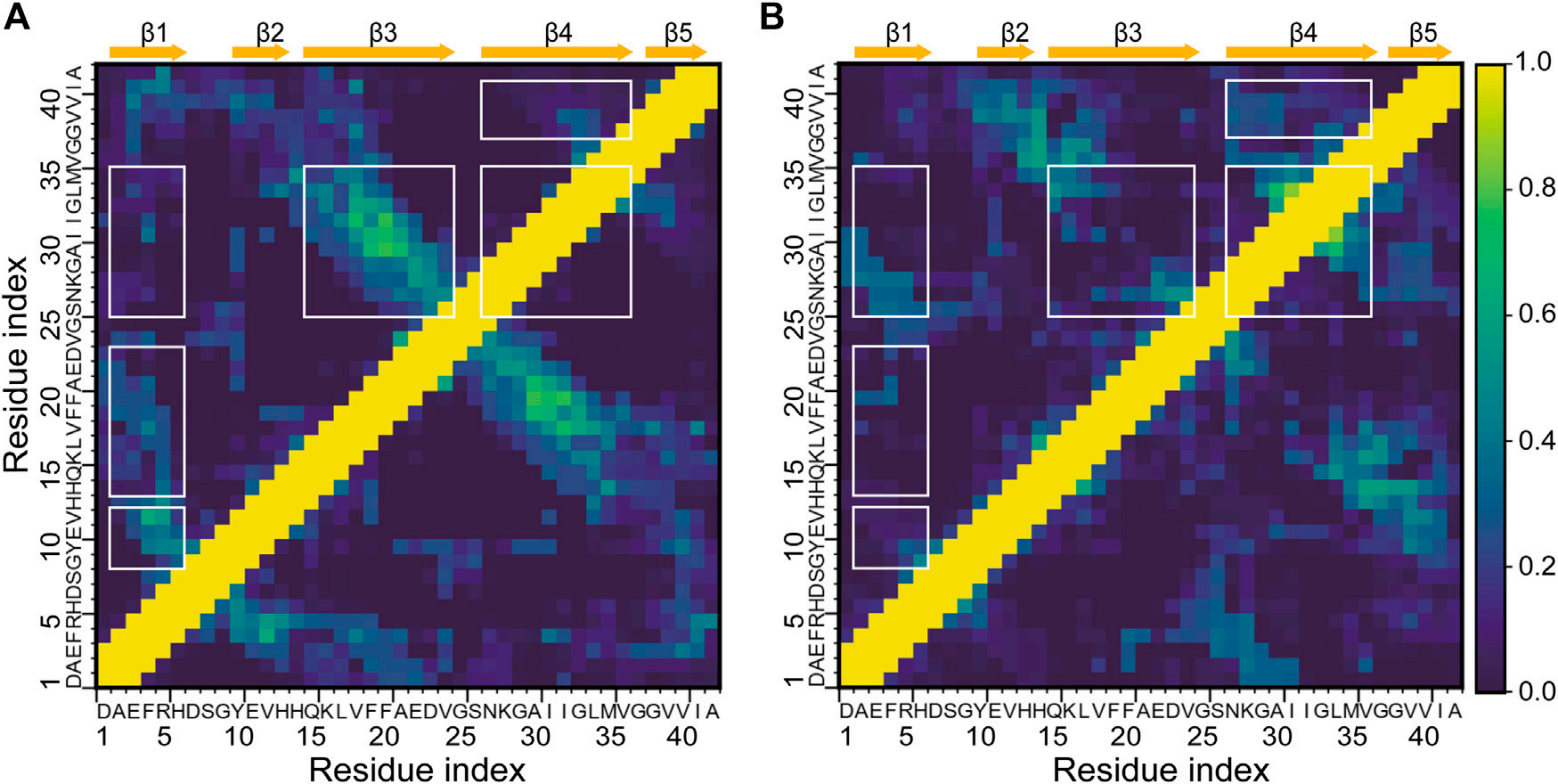
Intrapeptide interaction maps of the Aβ42 molecule **(A)** in the absence and **(B)** in the presence of HSA. Contact probabilities are displayed in a color scale from navy to yellow. Submatrices with distinct changes upon binding to HSA are highlighted by boxes in white.

The decrease in the abundance and lengths of β-strands together with the changes in β-sheet associations suggests that the intrapeptide interactions of Aβ42 would be changed by HSA. To validate this conjecture, we calculated the contact probabilities of all the residue pairs of Aβ42 with and without HSA and showed the results in Figure 4. For the isolated Aβ42, the matrix elements with high contact probabilities are away from the diagonal, indicating that long-range interactions are dominated. The strongest interactions are observed between Q15-V24 (β3) and N27-V36 (β4), consistent with the highest β-sheet propensities of β3 and β4 (Figure 1C) and the frequent association between the two (Figure 3B). The corresponding antidiagonal submatrix signifies an antiparallel arrangement of two β-strands, as observed in C1 and C3 to C6 (Figure 2A). The submatrix constituted by A2-H6 and Y10-H13 regions has the second highest contact probabilities, corresponding to the formation of β-hairpin by β1 and β2 in C4 and C6. The antidiagonal submatrix constituted by β1 and β3 regions also shows high contact probabilities, corresponding to antiparallel β-sheets in C1.

In the presence of HSA, interactions between β3 and β4 are greatly weakened, consistent with the decrease of β-sheet propensities at the two regions. Instead, β4-β5 interactions are enhanced as β4 frequently associates with β5 as well. Interactions of β1 with β2 and β3 are much weaker, too. The former results from the suppression of the β1-β2 associations, which only appears in C6′ with a population of 6.4%, as opposed to appearing in C4 and C6 of the isolated Aβ42 with a total population of 13.6%. The latter can be attributed to the disappearance of the β1-β3 association. In contrast, β1 is paired with β4 in C1′ and contributes to forming an antiparallel β-sheet. Consistently, the antidiagonal elements of the submatrix constituted by A2-R5 in β1 and S26-I31 in β4 display high contact probabilities. Lastly, local interactions within β4 are stronger, consistent with the β-sheet associations in C1′, where residues S26-I31 and L34-G37 within β4 are arranged into an antiparallel β-sheet.

The above results manifest that HSA interferes with the interactions of the Q15-V24 region with the A2-H6 and N27-V36 fragments, which are dominant in the isolated Aβ42 system and are essential for β-sheet formation. While such long-range interactions are prevented, local interactions within β4 and those between β4 and β5 are enhanced instead.

### Charged and Polar Residues in the N-Terminal Region and the K28-M35 Segment are More Likely to Interact With HSA

To explain the effect of HSA on Aβ conformations, next we analyzed the binding properties of Aβ42 with HSA. Clustering of the Aβ42 positions in all snapshots identifies five major binding poses (Supplementary Figure S4). Poses 1 and 4 are within domain III; pose 2 is at the cleft between domains I and III; poses 3 and 5 are within domain II. Obviously, domain III is the most populated binding site among the three HSA domains. Our previous work reported similar results (Guo and Zhou, 2019), wherein we attributed high binding propensities of domain III to its high conformational flexibility (Supplementary Figure S2B) which was essential for HSA to adapt Aβ binding. Here, we focus on the Aβ side.

**FIGURE 5:**
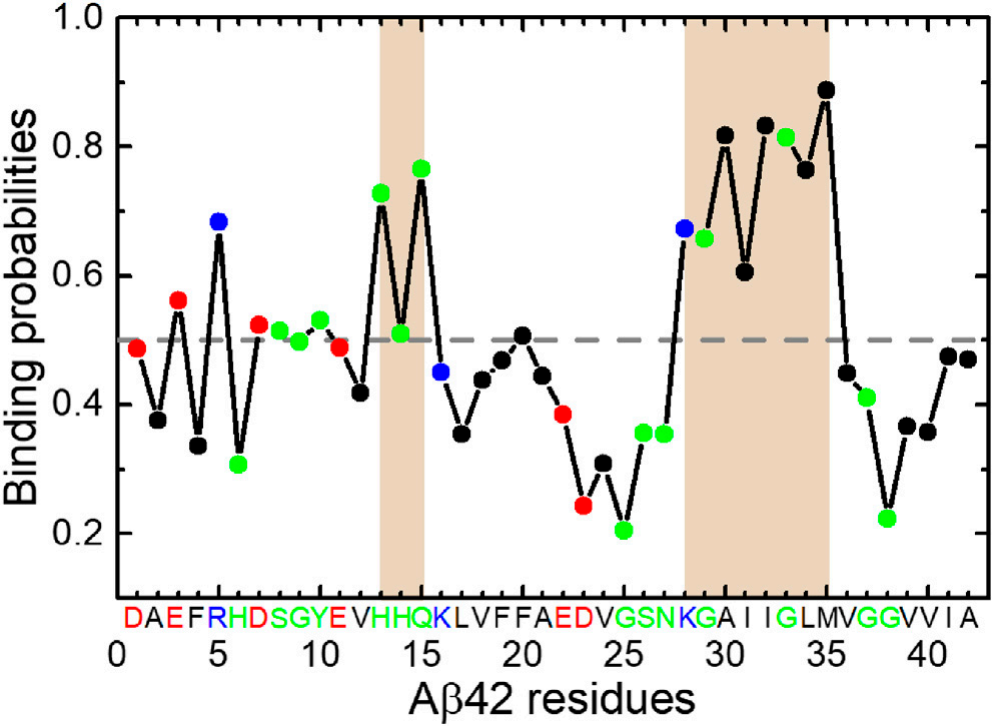
Binding probabilities of Aβ42 residues with HSA. Data points are colored according to residue types (acidic: red; basic: blue; polar: green; hydrophobic: black). Regions displaying relatively high binding probabilities are highlighted by brown shading. The average binding probability is displayed as a horizontal dashed line.

Interestingly the residue-specific HSA-binding probabilities of Aβ42 (Figure 5) show a dependence of residue types. In total, 20 residues have above-average binding propensities, among which 5 residues are charged, 9 are polar, and 6 are hydrophobic. The opposite trend is observed for the other 22 residues with below-average binding propensities. The number of charged, hydrophilic, and hydrophobic residues are 4, 5, and 13, respectively. It suggests that electrostatic interactions play an important role in Aβ42 binding to HSA.

The K28-M35 fragment has the highest binding propensities and is the primary interaction site with HSA. The central residues H13-Q15 also exhibit relatively high binding probabilities. These results are consistent with recent NMR data (Algamal et al., 2017) and our previous MD results (Guo and Zhou, 2019). Both studies have identified the C-terminal region as the primary binding site of HSA. It is noteworthy that the fragment K28-M35 is at the center of β4 region, which frequently associates with the Q15-V24 (β3) region into β-sheets in the absence of HSA. Although the β3 segment displays below-average HSA-binding probabilities, binding of the β4 segment to HSA would interfere with β3-β4 interactions and result in the loss of hydrogen bond partners for both. This result is reconciled with the decrease of β-sheet propensities at H14-E22 and A30-G33 regions and weaker interactions between Q15-V24 and N27-V36 regions. The C-terminal β-region V39-A42 exhibits below-average binding propensities, consistent with the NMR data (Algamal et al., 2017) which have shown that interaction of the C-terminal β-strand with HSA is reduced in Aβ42 monomer but promoted in protofibrils, possibly due to the stabilization of a C-terminal turn at G37 and G38 by the last two residues.

### Promiscuous Interactions Between Aβ42 and HSA Facilitate Optimal Binding but Disrupt Intramolecular Interactions Crucial for β-Sheet Formation

**FIGURE 6:**
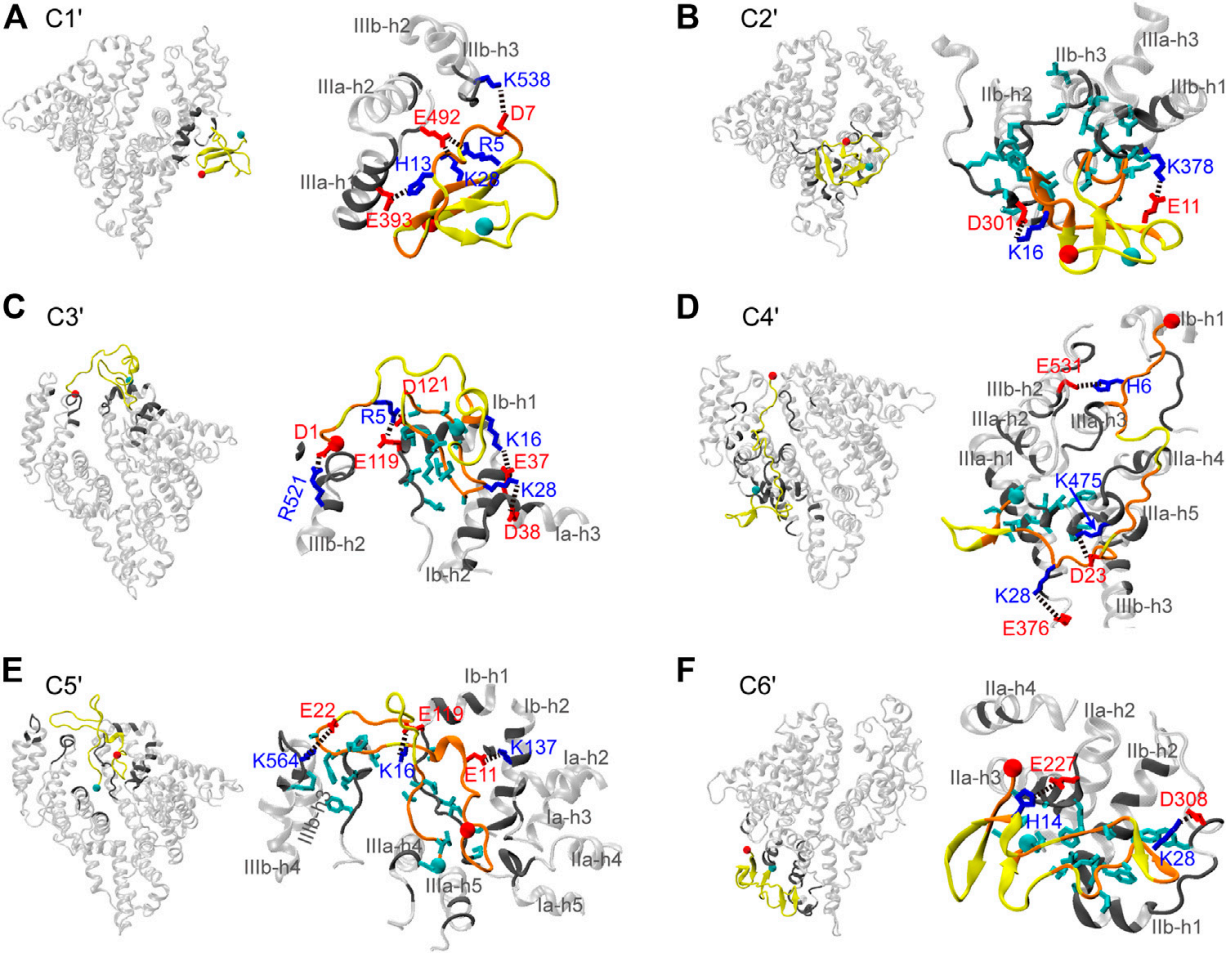
**(A–F)** Representative structures of the Aβ42-HSA complexes in the six most-populated clusters. In each panel, an overview of the complex is shown on the left and an enlarged view of the binding surface is shown on the right. HSA is transparent and residues in contact with Aβ42 are highlighted in gray. Aβ42 is in yellow and residues in contact with HSA are in orange. Side chains are colored according to the residue types (acidic: red; basic: blue; hydrophobic: cyan). The Cα atoms of residues D1 and A42 are indicated by red and cyan balls, respectively.

To further reveal the interaction mechanism of Aβ42 with HSA, structural characterizations of the Aβ42-HSA complex are necessary. By visual inspection of the snapshots, we found that the binding positions of Aβ42 are approximately the same among conformations of each cluster. Clusters C1′ to C6′ correspond to five binding poses, which are virtually identical to those shown in Supplementary Figure S4. The mapping relations between clusters and binding poses are as follows: C1′ to pose 1, C2′ to pose 3, C3′ and C5′ to pose 2, C4′ to pose 4, and C6′ to pose 5. Therefore, the corresponding complex conformations of cluster centers serve as good representations of all snapshots. Below we provide the structural details of each complex (Figure 6), paying special attention to potential conflict with intrapeptide interactions.

In C1′, the N-terminal (i.e., β1-β2) region and the K28-M35 fragment (i.e., β4) of Aβ42 bind to the pose enclosed by IIIa-h1, IIIa-h2, and the h2-h3 loop of IIIb (Figure 6A). These HSA-binding residues belong to a three-stranded β-sheet, which is lidded at the periphery of the complex by a random coil in the β3 region. At the edges of the binding interface, residues R5, D7, and H13 form salt bridges or hydrogen bonds (H-bonds) with HSA residues E492, K538, and E393, respectively, anchoring the Aβ42 peptide to HSA surface. Embedded within the complex, K28 forms a salt bridge with E492 of HSA, which positions the K28-M35 fragment in proximities of HSA. As a result, the β-sheet-rich structure of Aβ42 is trapped by HSA via direct interactions.

The binding pose of C2′ is constituted by the IIa-IIb loop, IIb-h3, IIb-h4, IIa-h1, IIa-h3, and IIa-h4 (Figure 6B). Aβ42 interacts with HSA mainly via residues H13-D23 (i.e., β3) and A30-V36 (i.e., β4). Just like in C1′, Aβ42 is anchored to HSA by two salt bridges (E11-HSA:K378 and K16-HSA:D301) at the edge of the interface. Interestingly, residues K16-F19 in the CHC region form an intermolecular β-sheet with HSA residues D301-S304. The hydrophobic loop (A30-V36) between two intramolecular β-sheets inserts into the hydrophobic groove between IIb-h3 and IIIa-h1, confining the β-sheets at the near side of HSA. At the far side, these β-sheets are covered by the disordered N-terminal residues. As can be seen, Aβ42 achieves optimal binding on the HSA surface via multipronged interactions including salt bridges, H-bonds, and hydrophobic stacking. The interaction pattern is independent of the binding sites and conformations of Aβ as manifested by preceding results and as detailed next. Intermolecular salt bridges and the corresponding probabilities are summarized in Supplementary Table S1.

C3′ and C5′ share a similar binding pose at the cleft between domains I and III, which involves Ia-h1, Ib-h1, Ib-h2, the Ia-Ib loop, IIIa-h3, IIIa-h4, IIIb-h1, IIIb-h2, IIIb-h3, and IIIb-h4 (Figure 6C,E). This binding site is similar to the one detected by mass spectroscopy (Choi et al., 2017). In two clusters, Aβ42 interacts with HSA via different residues but forms similar types of interactions. In C3′, several charged residues in the N-terminal region, S26-M35 (i.e., β4) and V39-A42 (i.e., β5) fragments are bound to HSA, whereas in C5′, all residues are in contact with HSA except the N27-L34 fragment (i.e., β4). In both clusters, hydrophobic residues (β4 and β5 for C3′ and β5 for C5′) are embedded into the groove surrounded by Ia-h3, Ib-h1, and Ib-h2, forming hydrophobic stacking with the Ia-Ib loop; charged residues form salt bridges at the interface boundaries, which involve D1, R5, K16, and K28 in C3′ and E11, K16, and E22 in C5′.

In C4′, Aβ42 binds to the backside of domains I and III, which involves the Ia-Ib loop, IIb-h3, IIb-h4, the entire IIIa, and the IIIa-IIIb loop (Figure 6D). It interacts with HSA extensively via residues D1-E11, L17-A21 (i.e., β3), D23-I31 (i.e., β4), and V39-A42 (i.e., β5). The N-terminal and central regions of Aβ42 are anchored to the HSA surface by salt bridges (D1-HSA:R114 and K28-HSA:E376) and H-bonds (e.g., H6-HSA:E531). Lastly, the binding pose of C6' is within domain II surrounded by IIa-h2, IIa-h3, IIa-h4, the IIa-IIb loop, IIb-h1, and IIb-h2 (Figure 6F). Residues D1-F4 (i.e., β1), V18-E22 (i.e., β3), S26-M35 (i.e., β4), and I41-A42 directly interact with HSA. Aβ42 adapts to the HSA surface via the K28-HSA:D308 salt bridge, H-bonds (e.g., H14-HSA:E227), and hydrophobic stacking of β3 and β4 regions with IIb-h2 and the IIa-IIb loop.

Both electrostatic and van der Waals interactions are at play in Aβ42 binding. The N-terminal residues, K16 and K28, contribute to forming salt bridges or H-bonds at the rim. Intermolecular H-bonds are especially prominent in C3′ and C4′ as listed in Table 1. Hydrophobic stacking via β4 or β5 regions is observed in most clusters. Generally, the electrostatic interactions are significantly stronger than the van der Waals interactions, except in C2′ and C5′ for which two terms are comparable to each other. In short, the Aβ42 peptide takes the advantage of intrinsic flexibilities to form promiscuous interactions with HSA at different binding sites. HSA usually directly targets the most occurring β-regions (i.e., β3 and β4), or it traps the β-sheet-rich conformation by protecting β-sheets from water. Either way, HSA interferes with the interaction determinants of Aβ42 aggregation.

**TABLE 1:** Hydrogen bonds and interaction potential energies between Aβ42 and HSA calculated for each cluster. Interaction energies are decomposed into the electrostatic (*E*_*elec*_) and van der Waals (*E*_*vdw*_) terms. Standard deviations are given in parentheses.

Cluster	C1′	C2′	C3′	C4′	C5′	C6′
Hydrogen bond	4.7 (2.1)	7.1 (2.3)	15.0 (3.1)	15.0 (3.1)	9.6 (2.4)	7.3 (2.1)
*E*_*elec*_ (kJ/mol)	−296 (97)	−342 (97)	−638 (146)	−782 (124)	−510 (93)	−494 (93)
*E*_*vdw*_ (kJ/mol)	−166 (37)	−374 (37)	−227 (37)	−560 (64)	−480 (44)	−283 (46)

## Discussion

We have applied the REST2 method to gain mechanistic insights into the interactions of Aβ42 with HSA through selectively enhanced sampling of the Aβ42 peptide. HSA dramatically changes the conformational ensemble of Aβ42 in several aspects. First, the suppression of overall β-sheet structures by HSA demonstrates the inhibitory effect on Aβ fibrillization. Second, conformations of Aβ42 are more disordered in the complex; long continuous β-strands (>6 residues) that are highly populated in the free state are completely impeded. Third, HSA weakens intrapeptide interactions and alters the patterns of remnant interactions as well. For the isolated Aβ42, the two most occurring β-regions Q15-V24 and N27-V36 assemble into β-sheets, serving as a core of β-sheet-rich structures. Residues A2-H6 interact strongly with residues Y10-H13 and Q15-V24. In the complex, all these interactions are impaired and new interaction pairs are formed. Residues Q15-V24 interact weakly with the rest of the peptide; residues N27-V36 switch to interact internally and with residues A2-H6 and V39-I41. For the other β-regions, β-sheet propensities are not affected by HSA, but interaction partners are different in the two systems.

Conformational changes of Aβ42 result from promiscuous interactions, which conflict with intramolecular β-sheet associations. HSA simultaneously interacts with both hydrophilic and hydrophobic regions, which mainly include the N-terminal charged and polar residues and the hydrophobic K28-M35 fragment. Two additional hydrophobic regions (CHC and C-terminus) directly interact with HSA as well but with lower probabilities. Electrostatic and van der Waals interactions cooperate to optimize the binding interface with the former being more dominant. The Aβ42-HSA interface is characterized by salt bridges or H-bonds primarily between the N-terminal region and HSA residues at the rim and stacking of hydrophobic regions at the center. Residues K16 or K28 adjacent to the hydrophobic core also form salt bridges with HSA in all six clusters. Consequently, interactions of the A2-H6 region with Y10-H13 and Q15-V24 regions are impaired; β-sheet probabilities and associations of Q15-V24 and N27-V36 regions are suppressed. In addition, such extensive interactions with HSA are incompatible with distal interactions. Instead, local intrapeptide interactions are preferable, such as interactions of the N27-V36 region with itself and the C-terminus.

Our findings provide atomistic insights into the role HSA played at the initial stage of Aβ aggregation. HSA could interfere with Aβ nucleation in several ways, which explains why HSA lengthens the lag phase of Aβ fibrillization (Stanyon and Viles, 2012). First, interactions with HSA hinder the β-sheet formation and eliminate structural characteristics resembling Aβ42 fibrils. In the free state, residues Q15-D23 and N27-V36 frequently associate into β-sheets as a core of β-sheet-rich conformations. Consistently, residues Q15-V18 and A30-I32 always formed β-sheets in all Aβ42 fibril structures (Supplementary Figure S5) (Xiao et al., 2015; Colvin et al., 2016; Wälti et al., 2016; Gremer et al., 2017). Residues H15-V24 formed a hydrophobic cluster with N27-L34, stabilizing a disease-relevant amyloid fibril (Wälti et al., 2016). However, HSA directly targets the second region (specifically K28-M35), significantly decreases the β-sheet abundance of both regions (especially H14-E22 and G30-G33), and impairs intrapeptide interactions between them. In additions, direct interactions of Aβ42 charged residues with HSA conflict with several salt bridges stabilizing fibril structures, including K28-D1 (Gremer et al., 2017), K28-A42 (Xiao et al., 2015; Colvin et al., 2016; Wälti et al., 2016), R5-D7 (Gremer et al., 2017), E11-H6 (Gremer et al., 2017), and E11-H13 (Gremer et al., 2017). Our findings are consistent with experiments by Stanyon and coworkers which have shown that Aβ bound to HSA is trapped in a nonfibrillar form (Stanyon and Viles, 2012). Second, although ordered β-sheet structures can be formed on the HSA surface, they are protected from exposure to water by HSA and disordered regions of Aβ42, which potentially prevents further β-sheet growth upon addition of monomers. Lastly, as Aβ42 binds to multiple sites on the HSA, it is possible that HSA concurrently traps several Aβ monomers, effectively decreasing the concentrations of monomers for nucleation. It is conceivable that HSA would interfere with Aβ42 dimerization by disrupting the common structural features shared by Aβ42 monomer and dimer (Man et al., 2017), which include similar β-sheet profiles, the β-hairpin spanning CHC and A30-V36 regions, and the three-stranded β-sheet involving L17-A21, A30-V36, and V39-I41. NMR data have shown that two terminal residues of Aβ42 extend direct interactions of protofibrils with HSA to the very C-terminal residues as compared to Aβ40 (Algamal et al., 2017). It would be interesting to carry out comparative simulations of multiple Aβ42 or Aβ40 monomers binding to HSA.

The promiscuity-centered interaction mechanism proposed here has important biological implication in the context of IDPs. Aβ42 and many other amyloid peptides (e.g., tau, amylin, and α-synuclein) belong to the family of IDPs. Accumulative evidence suggests that interacting partners of these IDPs can modulate the amyloidogenic process. In addition to diverse partners that interfere with Aβ fibrillization (Han et al., 2016; Wallin et al., 2017; Sun and Ding, 2020), amyloidosis of amylin is affected by 7B2, proSAAS (Peinado et al., 2013), lysozyme, and alpha-lactalbumin (Pilkington et al., 2017). IDPs usually promote nonspecific and dynamics multivalent interactions with targets (Weng and Wang, 2020). Some transition from disorder to order upon binding to protein partners while some keep various degrees of disorder. Aβ42-HSA interactions are typical of IDP-protein interaction regime. First, Aβ42 binds to multiple sites on the HSA surface and adopts multiple conformations including ordered β-sheet structures and extended random coils. Second, their interactions are promiscuous and do not rely on specific residue sequences. The binding interfaces commonly have salt bridges at the rim and hydrophobic cores at the center. In addition, electrostatic interactions known to enhance the binding rates and the formation of IDP-protein complexes (Zhou and Pang, 2018) play an important role in Aβ42 binding to HSA. The proposed Aβ42-HSA interaction mechanism reinforces the important role of promiscuous interactions in regulating amyloidosis. It might apply to other modulators of Aβ aggregation and probably is prevalent in the amyloid regulation by endogenous proteins.

Our study demonstrates that the REST2 method is suitable for studying IDPs, as other studies have done (Pang and Zhou, 2015; Rossetti et al., 2016; Smith et al., 2016; Han et al., 2017; Lee and Chen, 2017; Hicks and Zhou, 2018). More intriguingly, we present an example of using it to achieve efficient sampling of the IDP-protein complex, given that simulations of such systems are generally resource demanding. Our work has confirmed the theoretical expectation that the REST method can be readily used to only heat part of the system with affordable computational cost (Han et al., 2017) as the replica exchange probabilities exclusively depend on the degrees of freedom related to the hot region. For the isolated Aβ42 peptide, we obtained converged sampling with 16 replicas covering an effective temperature range from 300 to 600 K. Our results are consistent with previous REMD simulations, which in comparison used much more replicas for a similar temperature range (52 replicas spanning 270.0–601.2 K) (Rosenman et al., 2016). In the complex system, only Aβ42 was still chosen as the hot region as HSA experiences little conformational changes. Compared with the free monomer system, though the total number of atoms increases by an order of magnitude (∼16000 vs. ∼132000), the same number of replicas were used within the same temperature range. We hope that our study would spur future applications of REST2 on similar occasions, such as the binding and coupled folding of IDP to its globular protein partners whereby large-scale conformational changes occur to the IDP only.

## Data Availability

The raw data supporting the conclusion of this article will be made available by the authors, without undue reservation.
