# Supplementary material for: Albumin Alters the Conformational Ensemble of Amyloid-β by Promiscuous Interactions: Implications for Amyloid Inhibition
Source: Front Mol Biosci. 2021 Feb 23;7:629520. doi: 10.3389/fmolb.2020.629520 (PMC7940760; doi:10.3389/fmolb.2020.629520)
Supplement: Supplementary file 1 [file table1.docx]

**The supplementary materials include three figures.**


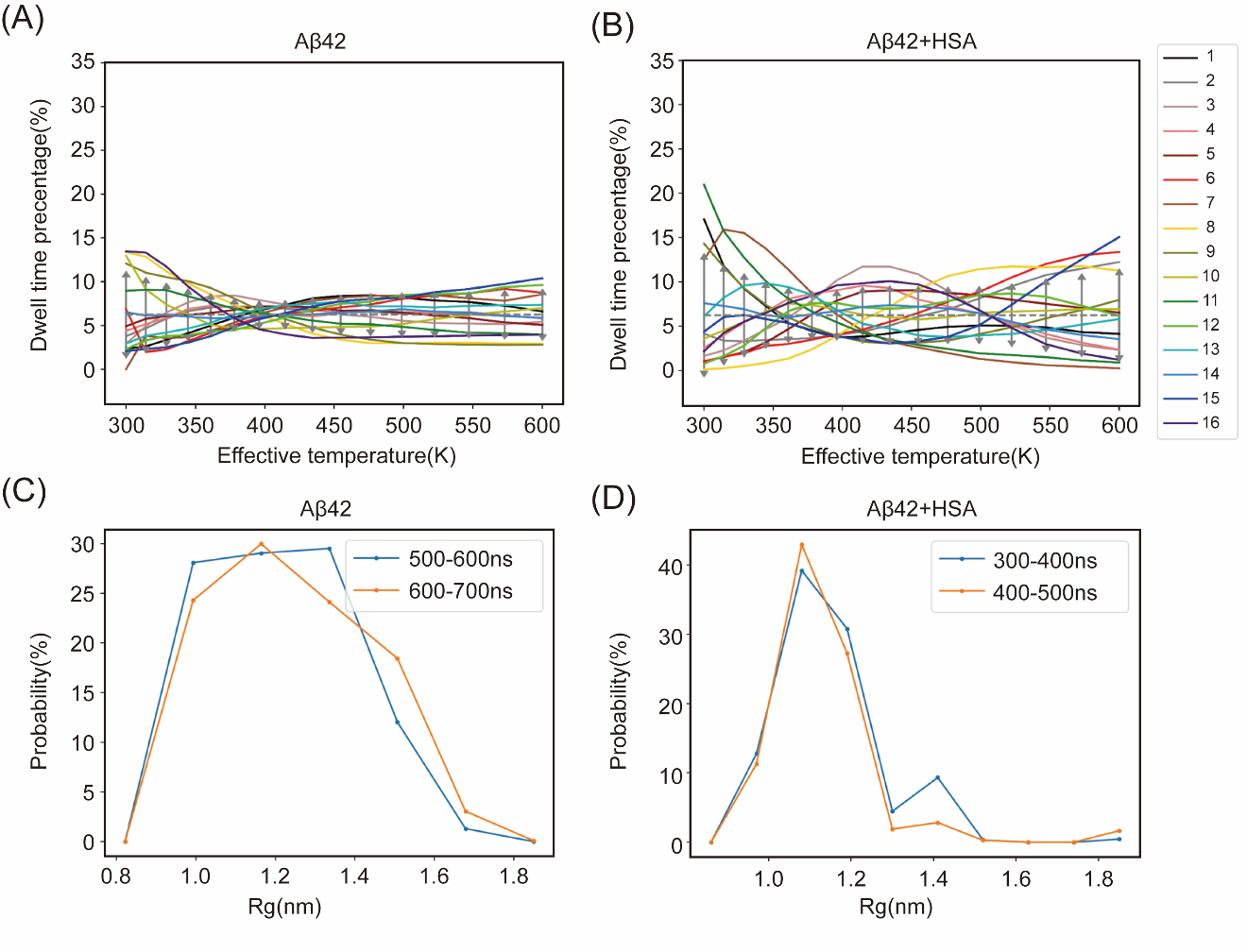


Figure S1. (A and B) The percentage of dwell time of 16 replicas at each effective temperature. The overall standard deviations are indicated by vertical arrow lines. (C and D) The distribution of the radius of gyration (Rg) of Aβ42 within two different time intervals from the unscaled replica.


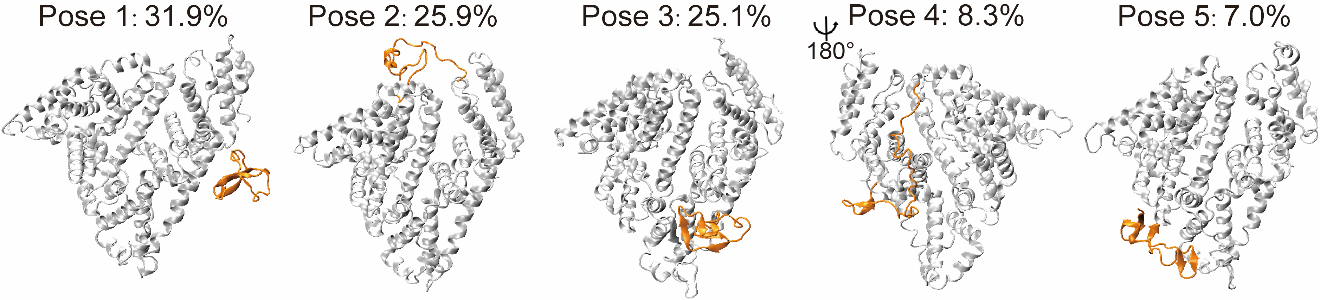


Figure S2. Clustering of the Aβ42 positions around HSA. Five major binding poses are found, which account for 98.2% of the total snapshots. For each pose, a representative snapshot is shown. HSA is in gray and Aβ42 is in gold.


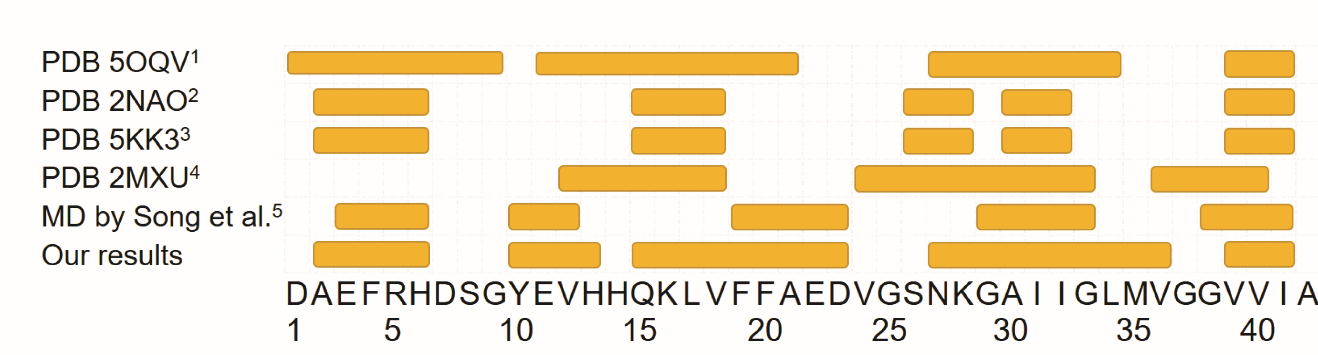


Figure S3. Comparison of β-regions identified by our simulations with those in previous simulations and Aβ42 fibril structures.

**References:**

1. Gremer, L., Schölzel, D., Schenk, C., Reinartz, E., Labahn, J., Ravelli, R. B. G., et al. (2017). Fibril structure of amyloid-β(1–42) by cryo–electron microscopy. *Science* 358, 116–119.
2. Wälti, M. A., Ravotti, F., Arai, H., Glabe, C. G., Wall, J. S., Böckmann, A., et al. (2016). Atomic-resolution structure of a disease-relevant Aβ(1-42) amyloid fibril. *Proc. Natl. Acad. Sci. U. S. A.* 113, E4976–E4984. doi:10.1073/pnas.1600749113.
3. Colvin, M. T., Silvers, R., Ni, Q. Z., Can, T. V., Sergeyev, I., Rosay, M., et al. (2016). Atomic resolution structure of monomorphic Aβ42 amyloid fibrils. *J. Am. Chem. Soc.* 138, 9663–9674. doi:10.1021/jacs.6b05129.
4. Xiao, Y., Ma, B., McElheny, D., Parthasarathy, S., Long, F., Hoshi, M., et al. (2015). Aβ(1-42) fibril structure illuminates self-recognition and replication of amyloid in Alzheimer’s disease. *Nat. Struct. Mol. Biol.* 22, 499–505. doi:10.1038/nsmb.2991.
5. Song, W., Wang, Y., Colletier, J. P., Yang, H., and Xu, Y. (2015). Varied probability of staying collapsed/extended at the conformational equilibrium of monomeric Aβ40 and Aβ42. *Sci. Rep.* 5, 1–13. doi:10.1038/srep11024.
