# Supplementary material for: Albumin Alters the Conformational Ensemble of Amyloid-β by Promiscuous Interactions: Implications for Amyloid Inhibition
Source: Front Mol Biosci. 2021 Feb 23;7:629520. doi: 10.3389/fmolb.2020.629520 (PMC7940760; doi:10.3389/fmolb.2020.629520)
Supplement: Supplementary file 2 [file table2.docx]

**The supplementary materials include one table and five figures.**

Table S1. Inter-molecular salt-bridges between Aβ42 and HSA residues and the corresponding probabilities in the top 6 clusters.

| Cluster | Aβ42 | HSA | Probability |
| --- | --- | --- | --- |
| C1' | R5 | E492 | 69% |
|  | D7 | K538 | 5% |
|  | K28 | E492 | 67% |
| C2' | E11 | K378 | 29% |
|  | K16 | D301 | 76% |
| C3' | D1 | R521 | 81% |
|  | R5 | E119 | 69% |
|  | R5 | D121 | 84% |
|  | K16 | E37 | 37% |
|  | K28 | D38 | 88% |
| C4' | D23 | K475 | 43% |
|  | K28 | E376 | 9% |
| C5' | E11 | K137 | 84% |
|  | K16 | E119 | 28% |
|  | E22 | K564 | 23% |
| C6' | K28 | D308 | 93% |


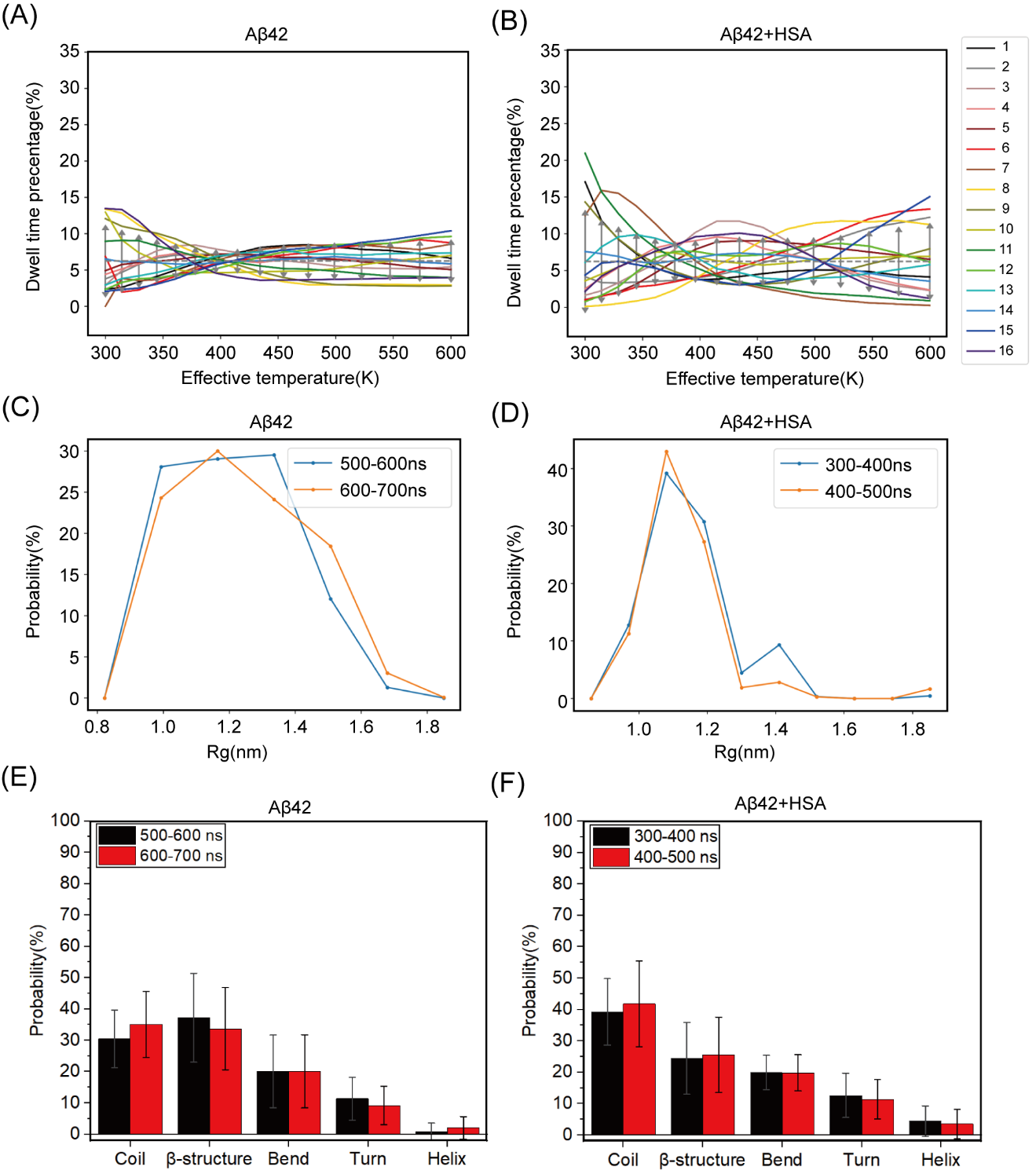


Figure S1. Convergence analysis for two systems. (A and B) The percentage of dwell time of 16 replicas at each effective temperature. The overall standard deviations are indicated by vertical arrow lines. (C and D) The distribution of the radius of gyration (Rg) of Aβ42 within two different time intervals from the unscaled replica. (E and F) Secondary structure probabilities of Aβ42 within two different time intervals from the unscaled replica.





Figure S2. Conformational changes of HSA at 300 K (blue curves), 414 K (yellow curves) and 600 K (red curves). (A) shows the time evolution of backbone RMSD of HSA. (B) shows the RMSF of all HSA residues. The average RMSF value is denoted by a horizontal dashed line. Regions with relatively high RMSFs are highlighted in brown shading.


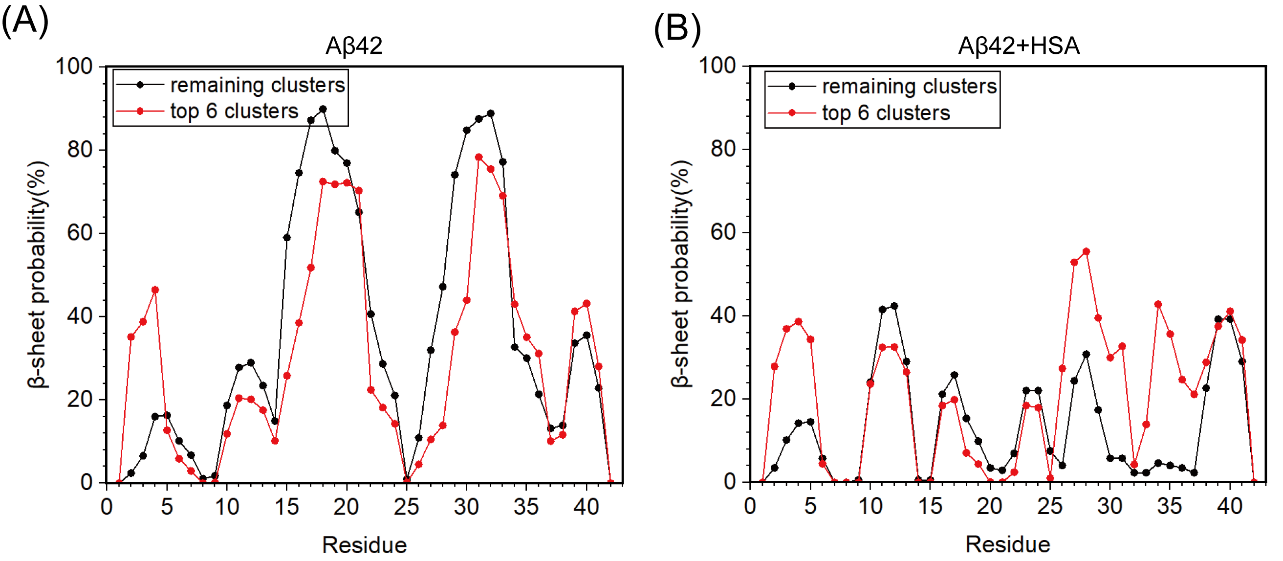


Figure S3. Residue-specific β-sheet probabilities of Aβ42 conformations sampled in the top 6 clusters and the remaining clusters in the (A) absence and (B) presence of HSA.


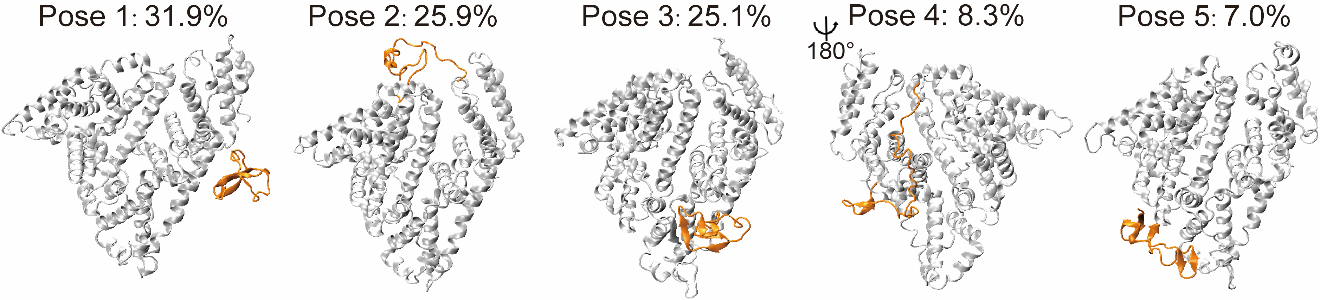


Figure S4. Clustering of the Aβ42 positions around HSA. Five major binding poses are found, which account for 98.2% of the total snapshots. For each pose, a representative snapshot is shown. HSA is in gray and Aβ42 is in gold.


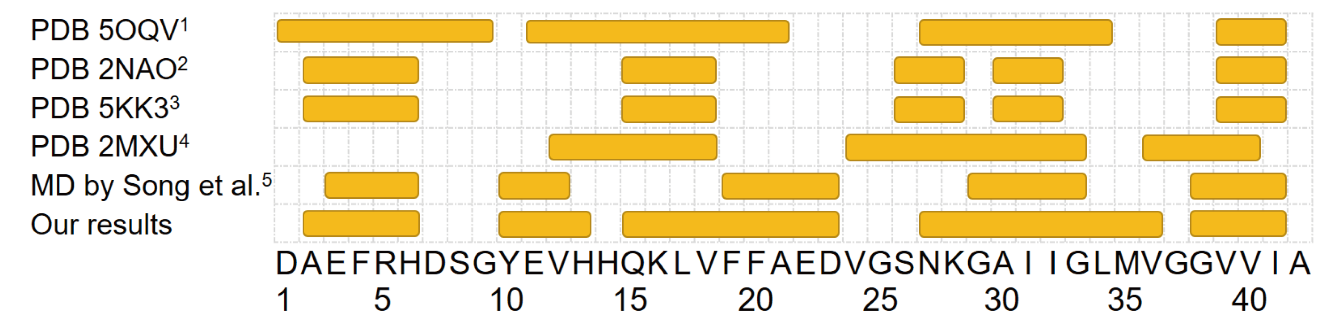


Figure S5. Comparison of β-regions identified by our simulations with those in previous simulations and Aβ42 fibril structures.

**References:**

1. Gremer, L., Schölzel, D., Schenk, C., Reinartz, E., Labahn, J., Ravelli, R. B. G., et al. (2017). Fibril structure of amyloid-β(1–42) by cryo–electron microscopy. *Science* 358, 116–119.
2. Wälti, M. A., Ravotti, F., Arai, H., Glabe, C. G., Wall, J. S., Böckmann, A., et al. (2016). Atomic-resolution structure of a disease-relevant Aβ(1-42) amyloid fibril. *Proc. Natl. Acad. Sci. U. S. A.* 113, E4976–E4984. doi:10.1073/pnas.1600749113.
3. Colvin, M. T., Silvers, R., Ni, Q. Z., Can, T. V., Sergeyev, I., Rosay, M., et al. (2016). Atomic resolution structure of monomorphic Aβ42 amyloid fibrils. *J. Am. Chem. Soc.* 138, 9663–9674. doi:10.1021/jacs.6b05129.
4. Xiao, Y., Ma, B., McElheny, D., Parthasarathy, S., Long, F., Hoshi, M., et al. (2015). Aβ(1-42) fibril structure illuminates self-recognition and replication of amyloid in Alzheimer’s disease. *Nat. Struct. Mol. Biol.* 22, 499–505. doi:10.1038/nsmb.2991.
5. Song, W., Wang, Y., Colletier, J. P., Yang, H., and Xu, Y. (2015). Varied probability of staying collapsed/extended at the conformational equilibrium of monomeric Aβ40 and Aβ42. *Sci. Rep.* 5, 1–13. doi:10.1038/srep11024.
